# Anesthetic Management of Resection of Metastatic Occipital Malignancy in a Patient With Fontan Physiology

**DOI:** 10.7759/cureus.18662

**Published:** 2021-10-11

**Authors:** Yusuf Mehkri, Geoffrey D Panjeton

**Affiliations:** 1 Department of Neurosurgery, University of Florida College of Medicine, Gainesville, USA; 2 Department of Anaesthesiology, University of Florida College of Medicine, Gainesville, USA

**Keywords:** anesthetic management, fontan physiology, perioperative mortality, open craniotomy, metastatic brain tumor

## Abstract

Significant advances in surgical management have allowed patients with congenital heart disease to survive to adulthood. Often, these patients present for non-cardiac surgeries, including patients who have undergone the three-staged Fontan repair for congenital single ventricle. The primary aim in the anesthetic management of adult patients with Fontan physiology is to maintain adequate venous pressure, low pulmonary vascular resistance (PVR) and normal contractility to maintain the cardiac output. We present the case of a 26-year-old female with Fontan physiology following a three-staged Fontan repair for tricuspid atresia who underwent a stealth-guided left occipital craniotomy for the palliative resection of a metastatic brain tumor.

This case highlights the importance of understanding Fontan physiology and its implications in the anesthetic management of a patient undergoing an open craniotomy. These patients require a high central venous pressure and low PVR to maintain optimum venous return to the left atrium. A rise in PVR can result in the shunting of the deoxygenated blood from the Fontan shunt to the systemic circulation. Hence, alpha agonists and high airway pressure are to be avoided. To minimize the risk of perioperative mortality, there is an increased need to optimize systemic to pulmonary blood flow ratios and maintain normal arterial saturation and euvolemic fluid status.

## Introduction

Globally, congenital heart disease (CHD) occurs in about 8-9 per 1000 live births, representing 28% of congenital defects in infancy [1]. Countless advancements have been made in the surgical management of CHDs over the last 30 years resulting in 85% to 90% of children born in the United States with congenital heart defects to survive to adulthood [2]. There are 800,000 to 1.2 million adults with treated CHD in the United States and it has been predicted that there will be soon more adult survivors with corrected CHD than children [3]. This group is referred to as grown-up with congenital heart disease (GUCHD) many of whom are likely to present for non-cardiac surgery, as demonstrated in the case report to follow. Anesthetic management of such cases requires a thorough understanding of the altered cardiovascular physiology, its implications on cardiac function and the impacts of anesthesia.

The incidence of single ventricle congenital anomaly is 2.3 per 10,000 live births and the incidence of tricuspid atresia is less than 1/10,000. The right ventricle is rudimentary or underdeveloped in tricuspid atresia and a large atrial septal defect (ASD) is required for the survival of these children and maintenance of cardiac output early in life. These children will be cyanotic because of the mixing of systemic venous blood and pulmonary venous blood in the common atrium. For the past four decades, the Fontan repair has remained the most commonly performed and effective procedure for patients with tricuspid atresia [4]. The main goal of this procedure is to separate the systemic and pulmonary circulations and to place them in series with the single functioning ventricle acting as a pump in a three-staged procedure.

Initially, a palliative surgery is performed to create a systemic to pulmonary shunt to improve oxygenation in the newborn. A Blalock-Taussig (right subclavian to the right pulmonary artery [PA]) shunt is one of the commonly done systemic to pulmonary shunts. In the second stage, anastomosis of the superior vena cava (SVC) to the main pulmonary artery (bidirectional Glenn) is performed. Finally, a total cavopulmonary shunt is achieved by directing the inferior vena cava (IVC) blood flow to the pulmonary artery. With the completion of the Fontan procedure, there is a single large atrium (a result of the existing ASD), supplying a single ventricle. As the venous blood is excluded from the right atrium, the cyanotic element is reduced, and these children typically maintain an oxygen saturation (SpO_2_) greater than 95%.

As a final step, a fenestration is created to redirect the blood from the venocaval shunt to the atrium and ventricle allowing to accommodate for a rise in systemic venous pressure and a decrease in venous return and cardiac output in the case of a rise in pulmonary vascular resistance (PVR). The fenestration will shunt the blood to the atrium and maintain the cardiac output at the cost of a diminished arterial oxygenation. Major modifications to the procedure that have decreased morbidity and mortality and improved cardiac output include staging the Fontan and using a fenestrated extra-cardiac conduit [5,6]. As Fontan patients enter adult life, in addition to cardiac issues such as arrhythmias and heart failure, they may also manifest conditions such as liver disease and psychiatric and neurocognitive issues [7,8]. For these patients, this once cardiovascular disease has become a multiorgan disease. As a result, knowledge of the appropriate anesthetic management of adult patients with CHD presenting for noncardiac surgery is of the utmost importance. The recently published literature indicates echocardiography guidelines and expert opinions reflecting that this group of patients is at risk of experiencing increased perioperative morbidity and mortality [2]. We are presenting a case report of the anesthetic management of a 26-year-old female patient with Fontan physiology who presented for a left occipital craniotomy for the resection of a metastatic brain tumor, followed by a review of Fontan physiology and its implication for anesthesia.

## Case presentation

A 26-year-old female with a history of CHD, tricuspid atresia, and single-ventricle physiology palliated by the Fontan procedure presented for left-sided stealth-guided occipital craniectomy for metastatic hepatocellular carcinoma (HCC). She was born with tricuspid atresia, moderate subaortic ventricular septal defect, and hypoplastic right ventricle and underwent multiple palliative procedures including PA band, Glenn, and fenestrated lateral tunnel Fontan. At the age of 10, she required permanent pacemaker placement for sinus node dysfunction. At the age of 25, she began to experience symptoms of abdominal pain, nausea, vomiting, and early satiety; abdominal CT imaging revealed a 7.5 x 10.4 x 11.4 cm enhancing left liver lesion and left portal vein tumor thrombus and later biopsy was consistent with HCC. Prior to proceeding with surgical resection, her pacemaker generator had reached end of life. Replacement was complicated by generator proximity to the surgical field and right femoral venous and right internal jugular venous occlusions identified periprocedurally. Subsequently, transvenous tempo-permanent single-chamber (atrial) pacemaker implant was performed to provide stability perioperatively. Preoperatively, she underwent embolization of vascular supply to the tumor with interventional radiology. Thereafter, she underwent uneventful primary surgical resection of the liver mass and cholecystectomy.

**Figure 1:**
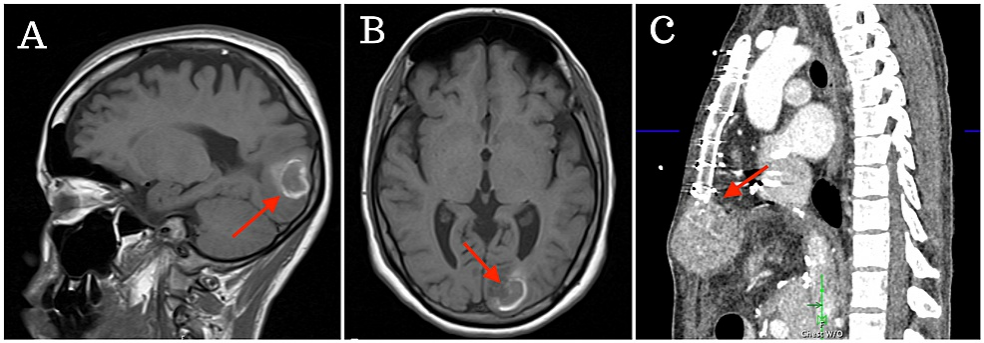
Imaging workup revealing metastatic HCC HCC, hepatocellular carcinoma (A) Sagittal view of MRI revealing a brain lesion consistent with hemorrhagic metastatic HCC; (B) axial view of MRI revealing an enhancing mass in the left occipital lobe; (C) CT of the chest and abdomen demonstrating a metastatic focus involving the lower sternum and xiphoid

Less than two months postoperatively, she developed sudden altered mental status presenting with slurred speech, right-sided eye deviation and tonic-clonic movements concerning for seizures. The initial CT imaging workup revealed a hemorrhagic lesion in the left occipital lobe with follow-up MRI also revealing a 2.5 x 2.2 x 2.3 cm cortical/subcortical enhancing mass centered in the left occipital lobe with a hemorrhagic component in the lateral aspect of the lesion with moderate adjacent vasogenic edema and sulcal effacement consistent with hemorrhagic metastatic HCC (Figure 1). CT imaging of the chest and abdomen reflected a complex solid lesion involving the lower sternum and xiphoid with interval growth and now appeared to have areas of internal necrosis, concerning for local metastasis and pulmonary nodules. She began to develop right-sided homonymous hemianopsia visual deficits and ultimately underwent stereotactic radiosurgery to address her occipital lesion. Additionally, she underwent five days of radiation therapy for her lower sternum/xyphoid mass and initiated chemotherapy with lenvatinib.

**Figure 2:**
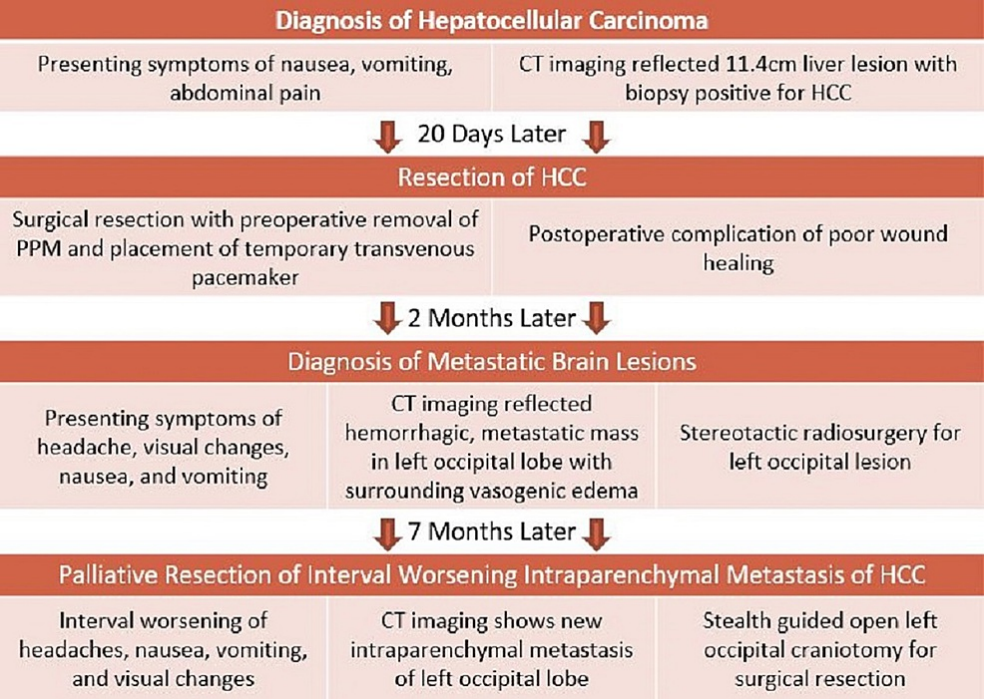
Patient history PPM, permanent pacemaker

Four months later, she developed recurrent palpitations and was diagnosed with a pulmonary embolus at an outside hospital and initiated rivaroxaban anticoagulation. The interval progression of her metastatic HCC included development of painful metastasis to her right posterior scapula, right parietal skull, and left distal femur. Palliative radiation therapy was performed for pain control in these areas. Radiation therapy treatment course was limited by the development of worsening headache, intractable nausea, and vomiting with worsening right-sided visual scotomas. Repeat brain MRI at this time revealed a new left lateral occipital lesion as well as an increase in the size of left medial occipital lesion. She was discharged at this time with a plan to return for stealth-guided left occipital craniotomy for the palliative surgical resection of the metastatic brain tumor to improve quality of life, and reduce symptom burden and prevent further visual loss following adequate time since the last dose of rivaroxaban (Figure 2).

Anesthetic management

In the preoperative holding area, the patient was awake, alert, and fully oriented to time, place, and person. She had a peripheral intravenous catheter placed by the nursing staff. Subsequently, a preoperative history and physical examination were performed by the anesthesia staff and the patient was taken to the operating room. Standard American Society of Anesthesiologists (ASA) monitors were placed and thereafter a right radial arterial line was placed under local anesthetic. Following a period of preoxygenation, the patient underwent intravenous induction of general anesthesia by initiating propofol infusion at 150 mcg/kg/min, and administering fentanyl 50 mcg, lidocaine 60 mg, etomidate 8 mg, and rocuronium 50 mg. Thereafter, the patient was mask ventilated and intubated without complication. General anesthesia was maintained using titrated doses of propofol (between 100-200 mcg/kg/min) and remifentanil (between 0.1-0.2 mcg/kg/min) infusions. A total intravenous anesthetic (TIVA) technique was preferred to an inhalational technique due to the decrease in the cerebral blood flow and metabolic rate while maintaining flow-metabolism coupling. Additionally, volatile anesthetics may have a greater degree of myocardial depression and a greater decrease in systemic vascular resistance as compared to intravenous anesthetics. As the planned surgical procedure was expected to last for three hours with minimal blood loss, we decided not to put a central line for central venous pressure (CVP) monitoring. Additionally, central venous access is not typically utilized for intracranial surgeries in our institution if adequate peripheral access can be obtained. Although CVP measurements can be helpful in guiding hemodynamics in patients with Fontan physiology, for this case in particular, the risks of central venous access were not outweighed by the potential therapeutic and diagnostic benefits. Levetiracetam and cefazolin were administered as requested by the surgical team. Mannitol was not administered for concern of hemodynamic tolerance of potential hypovolemia. Mild hyperventilation was maintained to a target end-tidal CO_2_ of 30-32 mmHg. The patient was placed in the three-quarter prone position with the left side up and the head was secured in the position desired for surgery with the three-point Mayfield frame.

The patient was hemodynamically stable and tolerated the procedure well. Mean arterial pressures ranged from 65 to 99 mmHg. SpO_2_ ranged from 89% to 98%, with the patient maintaining in the low 90s throughout much of the case consistent with her preoperative room air baseline measurement. Two arterial blood gas measurements were performed during the case, with the first exhibiting a PaCO_2_ of 44 mmHg with an EtCO_2_ of 30 mmHg, reflecting a significant alveolar-arterial (A-a) gradient. Ventilation parameters were adjusted and the subsequent PaCO_2_ measured 36 mmHg with an EtCO_2_ of 26 mmHg. Ventilation was controlled to a goal of 6 ml/kg tidal volume and peak inspiratory pressure ranging from 15 to 20 cm H_2_O with a respiratory rate of 16-20 breaths per minute.

Brief periods of hypotension following induction were managed with small bolus doses of phenylephrine and intravenous fluid administration. The total phenylephrine dose for the case was 100 mcg. Total fluid administration was 2 L and estimated blood loss was 250 ml. The total urine output was 1025 ml for a 4-hour and 25-minute total procedure time. Upon the conclusion of the procedure, neuromuscular blockade was reversed, and the patient was extubated and transported to the intensive care unit (ICU) eventfully. Postoperative neurological examination was normal.

The immediate postoperative course was complicated by bouts of emesis, consistent with preoperative baseline that improved with ondansetron and metoclopramide medications, and difficult to control headaches. She was transferred from the ICU to floor level care on postoperative day 2. On postoperative day 4, she was upgraded back to the ICU level of care following an episode of tachycardia with heart rate elevation to 220-230 beats per minute with a related decrease in the systolic blood pressure to the 60s and altered mental status that was responsive to adenosine and amiodarone administration. She was evaluated for concern of underlying atrial flutter and required further monitoring during medication titration. Ultimately, she was discharged home on postoperative day 10.

## Discussion

This case is unique in that appropriate anesthetic management is dependent on an adequate functional understanding of the physiologic implications of both open craniotomy and Fontan physiology. The patient was born with CHD involving tricuspid atresia, moderate subaortic ventricular septal defect, and hypoplastic right ventricle. These defects required a staged palliation, allowing the body to adjust to the various shifts in hemodynamic states and increasing the survivability of the underlying defect [9]. In order to appreciate the physiological implications of the Fontan operation, it is imperative to understand the intermediate stages of palliation. In the neonatal stage, a patient with tricuspid atresia will commonly undergo a procedure to create a systemic to pulmonary artery shunt resulting in a single ventricle pumping mixed blood to both the pulmonary and systemic circulations [10]. In this scenario, the balance of pulmonary and systemic blood determines the extent of cardiac output devoted to the systemic circulation. Often this procedure is paired with PA banding in order to maintain adequate systemic circulation by restricting the pulmonary blood flow [10]. The next of the staged procedures is typically performed in infancy and is known as the Glenn operation, wherein the superior vena cava is connected to the right pulmonary artery and divides the main pulmonary artery with the purpose of partially separating oxygenated and deoxygenated blood at the level of the ventricle [10]. This procedure is often delayed until three to nine months of life to allow for a decrease in PVR, that is necessary to allow for adequate flow through the shunt [2]. This has a dual physiological effect of decreasing the load on the single ventricle as the SVC return flows directly to the lungs, while maintaining preload as the IVC return to the single ventricle [10]. The final stage, typically performed in childhood, is known as the Fontan procedure of which a variety of configurations exist, but all ultimately completely separate the systemic and pulmonary circulations resulting in a passive, nonpulsatile flow from both the SVC and IVC to the pulmonary artery [10].

Fontan physiology is unique in that systemic venous pressure passively drives pulmonary circulation and although oxygenated blood does enter the heart through the left atrium as normal, oxygen saturation is rarely at 100% [11]. Two factors contribute to this relative hypoxemia: (1) small fenestrations are often created in the Fontan circuit to allow for pressure decompressing pop-off flow of the deoxygenated blood to flow into the atrium during times of increased pulmonary vascular resistance and (2) coronary sinus flow drains into the left atrium causing a slight desaturation [10,12]. This chronic relatively hypoxemic blood can have neurological consequences, including cognitive impairment, developmental delay, increased risk of neurological infection, seizures, and ischemia due to blood hyperviscosity [10]. The chronic hypoxemia is also related to decreased function of platelets, low activity levels of vitamin-K-dependent coagulation factors, factor V, and von Willebrand factor, implying that these patients may be at a higher bleeding risk [9].

The most pertinent anesthetic considerations when caring for patients with CHD are to (1) prevent arterial desaturation, (2) maintain systemic flow and pressure by maintaining systemic and pulmonary blood flow ratios, (3) minimize increases in PVR, and (4) ensure euvolemic fluid status [10]. Adequate monitoring is essential to safe and appropriate management of these patients. In addition to standard ASA monitoring, arterial line cannulation for beat-to-beat monitoring is typically used during neurosurgery and would be helpful in patients with CHD. It is important to note that Fontan physiology can also impact the interpretation of monitoring data. For example, decreases in SaO_2_ may indicate increases in pulmonary vascular resistance or decreased blood flow in Fontan circulation and the EtCO_2_ will underestimate PaCO_2_ due to the right-to-left shunt [10].

In the case of a craniotomy for the resection of brain tumor, goals of anesthetic management include decreasing intracranial pressure and brain relaxation. These goals are usually achieved using hyperventilation (typically titrated to EtCO_2_ in the low 30s), diuresis (typically with a weight-based bolus dose of mannitol), and positioning. All these interventions may be complicated in the presence of CHD (Table 1). Hyperventilation may lead to higher airway pressures resulting in increased intrathoracic pressures, which will result in increased pulmonary vascular resistance, decreased pulmonary blood flow, and alteration of the Qp/Qs ratio [10]. Similarly, diuresis-induced hypovolemia in a patient with Fontan physiology may result in a decrease in the transpulmonary gradient, decreasing pulmonary blood flow that would cause hypoxemia and systemic hypotension [10]. Some may consider administering diuretics and supporting hemodynamics with the inotropic agents; however, this may be ineffective in patients with Fontan physiology due to the limited influence of the heart on the cardiac output. In these instances, an alternative may be to place a lumbar drain, ensuring to consider anticoagulation status and the potential risks and benefits of the added procedure. Finally, elevating the head or placing the patient in a sitting position may decrease venous return that may upset the balance of Qp/Qs and adversely affect pulmonary blood flow [10]. Patients who have undergone a fenestrated Fontan have an artificially created communication between arterial and venous circulations that may allow for systemic and paradoxical embolus [2]. It is essential to meticulously ensure that all lines are adequately deaired while remaining vigilant for the possibility of air entraining through the venous sinuses [10].

**Table 1:** Management of craniotomy in the presence of CHD CHD, congenital heart disease; ICP, intracranial pressure

Intervention	Standard patient	Patient with CHD
Arterial cannulation	Adequate beat-to-beat monitoring	Increased significance
Hyperventilation	Reduces ICP and bleeding	Avoid pulmonary shunting due to hypocarbia
Diuresis	Reduces ICP and bleeding	Diuresis-induced hypovolemia may lower systemic blood flow
Three-quarter prone position	Ideal for occipital craniotomy	Does not adversely affect venous return

## Conclusions

With the increased survivability of congenital heart defects, more patients with CHD are presenting for noncardiac surgeries. These patients are at an increased risk of perioperative mortality. Neurosurgery specifically further increases the odds of perioperative mortality by fivefold compared to general surgery in adult patients with CHD. The presented case report is intended to serve as a reflection of some of the considerations to be made when caring for a patient with CHD who is presenting for a craniotomy.

## References

[1] Ntiloudi D, Giannakoulas G, Parcharidou D, Panagiotidis T, Gatzoulis MA, Karvounis H (2016). Adult congenital heart disease: a paradigm of epidemiological change. Int J Cardiol.

[2] Theruvath ID, Reeves ST (2015). Congenital heart disease in the adult presenting for noncardiac surgery. ASA Refresh Courses Anesthesiol.

[3] MacGillivray TE, Lin CH (2019). The growing number of adults surviving with congenital heart disease. Methodist Debakey Cardiovasc J.

[4] Driscoll DJ (2007). Long-term results of the Fontan operation. Pediatr Cardiol.

[5] d'Udekem Y, Iyengar AJ, Cochrane AD (2007). The Fontan procedure: contemporary techniques have improved long-term outcomes. Circulation.

[6] Saiki H, Kuwata S, Iwamoto Y (2019). Fenestration in the Fontan circulation as a strategy for chronic cardioprotection. Heart.

[7] Ohuchi H (2016). Adult patients with Fontan circulation: what we know and how to manage adults with Fontan circulation?. J Cardiol.

[8] Clift P, Celermajer D (2016). Managing adult Fontan patients: where do we stand?. Eur Respir Rev.

[9] Fredenburg TB, Johnson TR, Cohen MD (2011). The Fontan procedure: anatomy, complications, and manifestations of failure. Radiographics.

[10] Beheiry H El (2019). Neuroanesthesia and coexisting cardiac problems: congenital. In Co-Existing Diseases and Neuroanesthesia.

[11] D'souza S, Satarkar B, Bharne SS (2012). Anaesthesia for a minor procedure in a patient with fontan physiology. Indian J Anaesth.

[12] Gewillig M (2005). The Fontan circulation. Heart.

